# Spread of Respiratory Pathogens During the COVID-19 Pandemic Among Children in the Northeast of Italy

**DOI:** 10.3389/fmicb.2022.804700

**Published:** 2022-03-24

**Authors:** Carolina Cason, Giulia Zamagni, Giorgio Cozzi, Davide Tonegutto, Luca Ronfani, Chiara Oretti, Andrea De Manzini, Egidio Barbi, Manola Comar, Alessandro Amaddeo

**Affiliations:** ^1^Department of Advanced Translational Microbiology, Institute for Maternal and Child Health, IRCCS “Burlo Garofolo,” Trieste, Italy; ^2^Clinical Epidemiology and Public Health Research Unit, Institute for Maternal and Child Health, IRCCS “Burlo Garofolo,” Trieste, Italy; ^3^Emergency Department, Institute for Maternal and Child Health, IRCCS “Burlo Garofolo,” Trieste, Italy; ^4^Department of Services, Azienda Sanitaria Universitaria Integrata Giuliano Isontina (ASUGI), Trieste, Italy; ^5^Department of Medical Sciences, University of Trieste, Trieste, Italy

**Keywords:** SARS-CoV-2, respiratory tract infections, children, epidemiology, coinfections

## Abstract

The social distancing measures adopted during the coronavirus disease 2019 (COVID-19) pandemic led to a profound change in the behavioral habits of the population. This study analyzes the impact of restriction measures on the shaping of the epidemiology of common winter respiratory pathogens in the pediatric population of northeast of Italy. From August 2020 to March 2021, a total of 1,227 nasopharyngeal swabs from symptomatic pediatric patients were tested for the presence of severe acute respiratory syndrome coronavirus 2 (SARS-CoV-2), influenza A and B, adenovirus, other coronaviruses, parainfluenza virus 1–4, enterovirus, bocavirus, metapneumovirus, respiratory syncytial virus, rhinovirus, *Bordetella pertussis*, *Bordetella parapertussis*, and *Mycoplasma pneumoniae*. To relate virus positivity with the clinic characteristics of the subjects enrolled, multinomial logistic models were estimated. SARS-CoV-2 was detected in 5.2% of the children; fever resulted as risk factor for infection [relative risk ratio (RRR) = 2.88, *p* = 0.034]. Rhinovirus was detected in the 40.7% of the subjects, with cough and rhinitis as risk factors (respectively, RRR = 1.79, *p* = 0.001 and RRR = 1.53, *p* = 0.018). Other coronaviruses were found in 10.8% of children and were associated to pharyngodynia (RRR = 4.94, *p* < 0.001). Adenovirus, observed in 11.6% of subjects, showed to have fever as risk factor (RRR = 6.44, *p* < 0.001). Bocavirus was detected in 3.2% of children. In conclusion, our results showed that social isolation measures had an impact on the circulation of RSV and influenza, although children under the age of 2 were most affected by the other respiratory infections. Therefore, this study highlights the need for continuing surveillance for a delayed spread of RSV and other respiratory pathogens.

## Introduction

The coronavirus disease 2019 (COVID-19) pandemic originating in Wuhan, China, in December 2019, led to a profound change in the behavioral habits of the population. Worldwide, following the guidelines issued by the World Health Organization (WHO) [World Health Organization [WHO], 2020] and by European Centre for Disease Prevention and Control (ECDC) (Homepage European Centre for Disease Prevention and Control, 2020), restrictions were applied, aimed at containing severe acute respiratory syndrome coronavirus 2 (SARS-CoV-2) diffusion, which included case isolation, contact tracing, quarantine for contacts or following movements from high-risk areas, migration measures, up to the general lockdown (Khanna et al., 2020).

Italy was one of the countries most affected by the pandemic (Salute, 2020b) and the first among Western countries to impose a national lockdown starting from February 24, 2020 in conjunction with what has been defined as the first wave, with positive effects on the spread of the virus in the Italian regions (Signorelli et al., 2020). After a sharp reduction in cases in the summer of 2020, around October of the same year, the whole Europe experienced a second pandemic wave (Bontempi, 2021), while a third peak of cases occurred in Italy in March 2021.

The restrictions imposed by governments have also affected the pediatric population; specifically, the use of face masks was made mandatory starting from 6 years of age (Salute, 2020a), and access to schools has undergone profound limitations: the generalized lockdown at the beginning of the pandemic led to the physical closure of school activities at all levels, which gradually resumed, exclusively in telematic mode, starting from May 2020. From September 2020, the scholar activities have resumed in presence for 8 million students (Sebastiani and Palù, 2020), initially only for kindergarten and primary schools, and afterward for middle and high schools. In the first phase of the pandemic, children appeared to be less affected by SARS-CoV-2, with mild symptoms and lower hospitalization rates than adult cohorts (Bellino et al., 2020; Colson et al., 2020; Jones et al., 2020; Li et al., 2020; Garazzino et al., 2021). However, respiratory diseases are recognized as the most frequent cause of mortality and morbidity in infants and young children in the winter and spring period (Lanari et al., 2002; Zar and Ferkol, 2014) with about five to six infections annually (Chonmaitree et al., 2008). The pathogens most frequently associated with respiratory tract infections in children include human rhinovirus (HRV) (Vandini et al., 2019), respiratory syncytial virus (RSV) (Suleiman-Martos et al., 2021), influenza virus (Lafond et al., 2016), parainfluenza virus (Weinberg et al., 2009), adenovirus (Lynch et al., 2011), and also coronaviruses (Berry et al., 2015). Furthermore, 10% of respiratory diseases are caused by bacteria (Fahey et al., 1998). Recent studies conducted on children have reported a reduction, and in some cases the absence, in the detection of other respiratory viruses during the COVID-19 pandemic, particularly of influenza and RSV (Edwards, 2020; Kuitunen et al., 2020; Sullivan et al., 2020; Yeoh et al., 2020; Varela et al., 2021).

This prospective observational study analyzes the impact of restriction measures against SARS-CoV-2 by observing their effect on the epidemiology of common winter respiratory infections in the pediatric population in the northeast of Italy. Due to the lack of specificity of SARS-CoV-2 symptoms, a differential diagnosis with pediatric diseases occurring in the winter and spring months is difficult in children (Fantini et al., 2020; Yasuhara et al., 2020). For this purpose, a new test for the surveillance of respiratory viruses in nasopharyngeal swabs was introduced, capable of detecting 13 of the most common pathogens of the respiratory tract (multiplex PCR). The virus positivity detected was also related to the demographic and clinical characteristics of the subjects enrolled.

## Materials and Methods

### Study Population and Data Collection

In this observational study, all subjects from 0 to 17 years old who had multiplex PCR from nasopharyngeal swab for the research of respiratory pathogens, at the tertiary-level university Maternal and Child Hospital IRCCS Burlo Garofolo (Trieste, Italy) from August 1, 2020 to March 31, 2021, were enrolled. Personal data collection included age, sex, symptoms, contact to subject positive of SARS-CoV-2, ward of provenience, and result of the test. Patients were divided into four different age groups: 0–2, 2–5, 6–9, and 10–17 years old. The age stratification was set on the basis of the Italian school system. Under the age of 2, children are cared for exclusively at home or attend prekindergarten; from 2 to 5 years of age, attend kindergarten; from 6 to 9 years of age, attend primary school; and after 10 years of age, attend middle and high schools.

Multiplex PCR was performed in case of symptoms attributable to respiratory infection (fever, chills, fatigue, muscle aches, pharyngodynia, cough, rhinitis, otalgia, dyspnea, bronchospasm, chest pain, headache, abdominal pain, nausea/vomiting, diarrhea, loss of taste, anosmia, skin lesions, and conjunctivitis) and/or contact with SARS-CoV-2-positive subject. The test was requested by the pediatric emergency room (PER) of the hospital, by an inpatient ward, or by family pediatricians or general practitioners (GP).

### Genetic Material Extraction

The genetic material extraction was performed from nasopharyngeal swabs starting from 200 μl of the samples at a final elution volume of 50 μl, using the automatic extractor Maxwell RSC Viral TNA Kit (Promega, Madison, WI, United States), according to the manufacturer’s instruction. The efficiency of the extraction kit was tested for both viral and bacterial strains and positive controls included in the assay, examined through multiplex PCR and reverse hybridization, with correct identification of all the microorganisms.

### Microbial Characterization Through Multiplex PCR and Reverse Hybridization

The identification of pathogens causing acute respiratory infections was performed using a multiplex PCR followed by reverse dot blot automatic hybridization into a macroarray CHIP based on DNA-Flow Technology (Hybri-Spot) (Vitro Master Diagnostica, Seville, Spain). The assay is based on two parallel multiplex PCR amplifications of each sample, optimized using 5 μl of genetic material for each reaction, with biotinylated primers followed by an automatic reverse hybridization in the membrane containing specific probes for detecting the most important microorganism associated with infections of the respiratory tract. Positive signals are visualized *via* a colorimetric immunoenzymatic reaction in a CHIP membrane by the Hybri-Spot platform (HS12) (Vitro Master Diagnostica, Seville, Spain). The colorimetric reaction is captured as image by a camera and analyzed by the Hybri-Soft software reporting the pattern of positive signals. The assay can simultaneously detect the presence of 13 pathogens: influenza virus type A (subtype H3 and H1N1) and type B, adenovirus (types 1–4, 6–8, 11, 12, 16, 18, 21, 31, 34), bocavirus, SARS-CoV-2, other coronaviruses (including coronavirus 229E, HKU-1, NL63, and OC43), metapneumovirus, human parainfluenza virus types 1–4 (HPIV), RSV subtypes A and B, HRV, enterovirus (EV-A, EV-B, and EV-D), *Bordetella pertussis*, *Bordetella parapertussis*, and *Mycoplasma pneumoniae*. The analytical specificity of the test is 100% for all the microorganisms included. The analytic limit of detection varies from 10 to 1,000 copies/reaction depending on the organism.

### Statistical Analysis

The categorical variables were reported as absolute and percentage frequencies. Fisher’s exact test was used to evaluate the hypothesis of independence in distribution. Continuous variables were reported as mean and standard deviation (SD). To identify the characteristics and symptoms associated with an increased risk of positivity to each of the viruses considered, bivariate logistic models were estimated. In addition, bivariate logistic regression was also used in order to evaluate the effect of age on the risk of presenting any coinfection. The multivariate analysis was carried out using a multinomial logistic model considering the negative result of the swab as the reference category: subjects with coinfections, asymptomatic, and all those from departments other than the PER were excluded. The significance level was set at 0.05. The statistical analysis was carried out with the software StataCorp (2021, Stata Statistical Software: Release 17, College Station, TX, United States: StataCorp LLC).

## Results

### Description of Cohort

From August 2020 to March 2021, a total of 1,227 children, 670 male (54.6%) and 557 female (45.4%), got access to the Maternal and Child Hospital IRCCS Burlo Garofolo in Italy to be tested for respiratory pathogens of the upper airways, including SARS-CoV-2. The demographic characteristics of the children enrolled in the study are summarized in Table 1. The children were thus distributed by age: 299 < 2 years old (24.4%), 462 between 2 and 5 years old (37.6%), 201 between 6 and 9 years old (16.4%), and 265 from 10 to 17 years old. Most of the samples tested came from children afferent to the PER (666, 54.3%) or addressed by GP (536, 43.7%); only 25 subjects (2%) were from other hospital wards. For 51 subjects (4, 2%), a request was made for multiplex analysis of respiratory pathogens following contact with a person who tested positive for SARS-CoV-2.

**TABLE 1:** Characteristics of children enrolled in the study.

Total	*N* = 1,227
	
Age (years)	*N* (%)
<2	299 (24.4)
2–5	462 (37.6)
6–9	201 (16.4)
10–17	265 (21.6)
**Sex**	
Male	670 (54.6)
Female	557 (45.4)
**Ward of provenience**	
PER	666 (54.3)
GP	536 (43.7)
Other	25 (2.0)
**Contact with positive person to SARS-CoV-2**	
No	1,176 (95.8)
Yes	51 (4.2)

*PER, pediatric emergency room; GP, general practitioner.*

As shown in Table 2, 61% of subjects confirmed positivity to an infection, the majority to HRV (499, 40.7%), followed by adenovirus (142, 11.6%), other coronaviruses (133, 10.8%), SARS-CoV-2 (64, 5.2%), and bocavirus (39, 3.2%). Four subjects resulted positive for *Mycoplasma pneumoniae* (0.3%). Few children resulted positive for the other microorganisms considered: HPIV (3, 0.2%), *Bordetella parapertussis* (2, 0.2%), enterovirus (2, 0.2%), metapneumovirus, (2, 0.2%), and RSV (1, 0.1%). The presence of influenza virus or *B. pertussis* was found in none of the samples analyzed in the study.

**TABLE 2:** Number of positive subjects for each pathogen researched.

Results of analyses	*N* (%)
Negative	465 (37.9)
Positive	762 (61.1)
**Pathogens detected**	
SARS-CoV-2	64 (5.2)
HRV	499 (40.7)
Adenovirus	142 (11.6)
Other coronaviruses	133 (10.8)
Bocavirus	39 (3.2)
*Mycoplasma pneumoniae*	4 (0.3)
HPIV	3 (0.2)
*Bordetella parapertussis*	2 (0.2)
Enterovirus	2 (0.2)
Metapneumovirus	2 (0.2)
RSV	1 (0.1)
Influenza virus	0 (0%)
*Bordetella pertussis*	0 (0%)

*HRV, human rhinovirus; HPIV, human parainfluenza virus; RSV, respiratory syncytial virus.*

### Descriptive Statistics by Age Group

The characteristics of the children enrolled, divided by age group, are described in Table 3. Age distribution of enrolled subjects by ward of provenance showed statistical significance (*p* < 0.001). Most children from the PER were over 2 years old (44%, 2–5 years; 53.7%, 6–9 years; 55.5%, 10–17 years), while children under 2 years of age were more frequently referred by the GP (216/299, 72.2%). For all age groups considered, the percentage of subjects who had contact with a SARS-CoV-2-positive person was below 6%. Virus positivity was distributed as follows: 206/299 (68.9%) under 2 years of age, 319/426 (69.1%) between 2 and 5 years, 112/201 (55.7%) between 6 and 9 years, and 125/265 (47.2%) in the age group 10–17. There was a significantly different distribution according to the age groups, SARS-CoV-2 was more frequent (*p* = 0.014) in children under the age of 2 than in those of different ages; other coronaviruses were more frequent (*p* < 0.001) in children under the age of 5, and adenovirus (*p* < 0.001). On the contrary, bocavirus was more frequent (*p* < 0.001) in the age group 2–5 years, while it was not found in any child over 10 years old.

**TABLE 3:** Descriptive statistic by age group.

	Age (years)				
	<2 *N* = 299	2–5 *N* = 462	6–9 *N* = 201	10–17 *N* = 265	*p*-value
**Ward of provenience, *N* (%)**					**<0.001**
PER	78 (26.1)	203 (44.0)	108 (53.7)	147 (55.5)	
GP	216 (72.2)	251 (54.3)	88 (43.8)	111 (41.9)	
Other	5 (1.7)	8 (1.7)	5 (2.5)	7 (2.6)	
**Contact with positive person to SARS-CoV-2**					0.379
No	287 (96.0)	445 (96.3)	195 (97.0)	249 (94.0)	
Yes	12 (4.0)	17 (3.7)	6 (3.0)	16 (6.0)	
**Results of analyses**					**<0.001**
Negative	93 (31.1)	143 (30.9)	89 (44.3)	140 (52.8)	
Positive for some infection	206 (68.9)	319 (69.1)	112 (55.7)	125 (47.2)	
**Pathogens detected**					
SARS-CoV-2	25 (8.4)	14 (3.0)	10 (5.0)	15 (5.7)	**0.014**
Other coronaviruses	44 (14.7)	66 (14.3)	16 (8.0)	7 (2.6)	**<0.001**
HRV	105 (35.1)	207 (44.8)	84 (42.8)	103 (38.9)	0.056
Adenovirus	63 (21.1)	68 (14.7)	6 (3.0)	5 (1.9)	**<0.001**
Bocavirus	5 (1.7)	30 (6.5)	4 (2.0)	0	**<0.001**
HPIV	2 (0.7)	1 (0.2)	0	0	0.445
Others	2 (0.7)	6 (1.3)	0	3 (1.1)	0.370

*PER, pediatric emergency room; GP, general practitioner; HRV, human rhinovirus; HPIV, human parainfluenza virus.*

*Others included: Mycoplasma pneumoniae, Bordetella parapertussis, enterovirus, metapneumovirus, RSV, influenza virus, and Bordetella pertussis. Significant p-values are highlighted in bold.*

### Temporal Positivity to Respiratory Pathogens (August 2020–March 2021)

**FIGURE 1:**
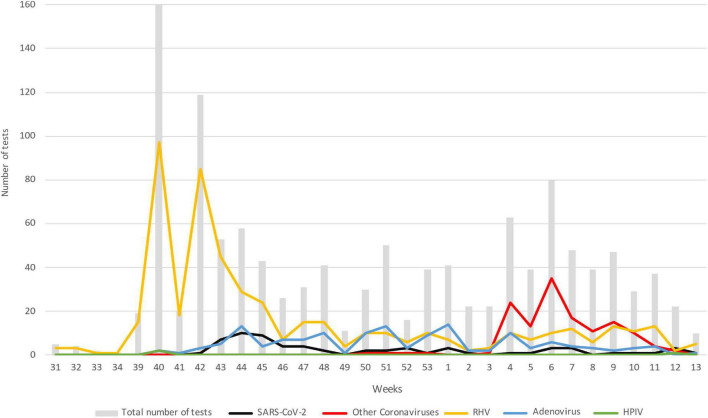
Trend in the number of the main pathogens detected over time; data are represented as the number of positive subjects for each of the viruses considered: gray bar, total number of test; black, SARS-CoV-2; red, other coronaviruses; yellow, HRV; blue, adenovirus; green, HPIV. Data are represented starting from August 1, 2020 (week 31) until March 31, 2021 (week 13). HRV, human rhinovirus; HPIV, human parainfluenza virus.

Figure 1 describes the trend over time of the main pathogens detected, divided by week starting from the collection of the first sample, on August 1, 2020 (31st week of the year) until March 31, 2021 (week 13). To note, while SARS-CoV-2 maintained values below 10 cases per week, with a maximum around the 44–45th week of 2020, HRV had two peaks of positivity at the 40th and 42nd week of 2020 maintaining consistently higher values than SARS-CoV-2. The growth trend of the other coronaviruses instead started from the third week of 2021, in January, until reaching the highest point in the sixth week of the same year and decreasing around the 12th week, corresponding to mid-March. The detection of adenovirus instead took place starting from the 41st week of 2020 with values always lower than 18 weekly cases.

### Coinfections

Of the subjects analyzed, 116 out of 1,227 (9.5%) had coinfections, as detailed in Table 4. To note, most of the coinfections were represented by adenovirus/HRV, detected in 49 subjects out 116 (42.2%) followed by HRV/other coronaviruses (19/116, 16.4%), HRV/bocavirus (14/116, 12.1%), SARS-CoV-2/HRV (11/116, 9.5%), and adenovirus/other coronaviruses (9/116, 7.8%). Simultaneous positivity to three pathogens was reported for only six subjects. The results of the bivariate logistic model for the risk of infections by age group is reported in Table 5. Children younger than 2 years old were considered as reference category; compared to these subjects, the risk of coinfections was not significantly different for those of age between 2 and 5 years old. However, age appeared to be a protective factor on the risk of having any coinfection. For subjects 6–9 years old, there was a reduction in the risk of coinfections over 70% (OR = 0.28, *p* = 0.002), reaching 87% (OR = 0.13, *p* < 0.001) for children over 10 years old.

**TABLE 4:** Coinfections detected.

Coinfections detected (total: 116/1,227, 9.5%)	*N* (%)
SARS-CoV-2/other coronaviruses	1 (0.9)
SARS-CoV-2/HRV	11 (9.5)
SARS-CoV-2/adenovirus	0
SARS-CoV-2/bocavirus	3 (2.5)
HRV/other coronaviruses	19 (16.4)
HRV/adenovirus	49 (42.2)
HRV/bocavirus	14 (12.1)
Adenovirus/other coronaviruses	9 (7.8)
Adenovirus/bocavirus	0
Bocavirus/other coronaviruses	3 (2.5)
SARS-CoV-2/HRV/adenovirus	1 (0.9)
SARS-CoV-2/adenovirus/bocavirus	1 (0.9)
HRV/adenovirus/bocavirus	2 (1.7)
HRV/adenovirus/enterovirus	1 (0.9)
HRV/other coronaviruses/bocavirus	2 (1.7)

*HRV, human rhinovirus.*

**TABLE 5:** Bivariate logistic model for the risk of coinfections by age group.

Coinfections	OR	95% CI	*p*-value
**Age (years)**			
<2 (ref.)	–	–	–
2–5	1.12	(0.73; 1.73)	0.592
6–9	0.28	(0.13; 0.62)	**0.002**
10–17	0.13	(0.05; 0.34)	**<0.001**

*Significant p-values are highlighted in bold.*

### Risk Factors

Having the dependent variables considered in the study with more than two nominal categories (0 = negative swab, 1 = SARS-CoV-2, 2 = other coronaviruses, 3 = HRV, 4 = adenovirus, and 5 = bocavirus), a multinomial logistic model was applied for the positivity to one of the five viruses, considering the negative test as the reference category. Those found to be significant in the bivariate analysis were included as independent variables. The model was estimated on 1,035 observations and coinfections, and the departments of origin other than PER or GP were removed. Results are reported in Table 6. For SARS-CoV-2, the major risk factor was a contact with a positive person to the same virus, with a relative risk ratio (RRR) of 17.25, *p* < 0.001. Fever was also a risk factor (RRR = 2.88, *p* = 0.034). For other coronaviruses, an age-protective effect emerged: as 1 year of age increased, the subject was expected to test negative rather than positive for this virus. The PER as department of origin, on the other hand, had a RRR = 2.8; therefore, those coming from the PER had a greater probability of being classified as positive to other coronaviruses, rather than being negative. The same emerged for pharyngodynia (RRR = 4.94, *p* < 0.001) and cough (RRR = 3.13, *p* < 0.001). Cough (RRR = 1.79, *p* = 0.001) and rhinitis (RRR = 1.53, *p* = 0.018) were risk factors for HRV, while age (RRR = 0.95, *p* < 0.001), origin from PER (RRR = 0.40, *p* < 0.001), and fever (RRR = 0.65, *p* < 0.009) were protective factors. Fever and origin from PER were, on the contrary, risk factors for positivity to adenovirus (respectively, RRR = 2.14, *p* = 0.025 and RRR = 6.44, *p* < 0.001); for this virus instead, age was found to be a protective factor (RRR = 0.78, *p* < 0.001). Finally, for bocavirus, there were no significant effects of the variables on the risk of being positive for this infection.

**TABLE 6:** Multinomial logistic model for the positivity to one of the detected viruses.

Ref. Negative swab	SARS-CoV-2			Other coronaviruses			HRV			Adenovirus			Bocavirus		
					
	RRR	95% CI	*p*-value	RRR	95% CI	*p*-value	RRR	95% CI	*p*-value	RRR	95% CI	*p*-value	RRR	95% CI	*p*-value
Age	0.98	(0.90; 1.06)	0.539	0.85	(0.79; 0.91)	**<0.001**	0.95	(0.92; 0.99)	**0.008**	0.78	(0.71; 0.86)	**<0.001**	0.88	(0.75; 1.03)	0.112
Ward of provenience															
GP (ref.)	–	–	–	–	–	–	–	–	–	–	–	–	–	–	–
PER	1.76	(0.75; 4.11)	0.193	3.13	(1.65; 5.93)	**<0.001**	0.40	(0.29; 0.55)	**<0.001**	2.14	(1.10; 4.18)	**0.025**	2.81	(0.57; 13.8)	0.201
Contact with positive person to SARS-CoV-2	17.25	(4.90; 60.71)	**<0.001**	–	–	–	1.22	(0.33; 4.44)	0.768	0.82	(0.09; 7.72)	0.864	–	–	–
Fever	2.88	(1.08; 7.69)	**0.034**	1.18	(0.69; 2.03)	0.546	0.65	(0.47; 0.90)	**0.009**	6.44	(2.56; 16.20)	**<0.001**	0.44	(0.12; 1.67)	0.228
Pharyngodynia	0.99	(0.28; 3.53)	0.991	4.94	(2.60; 9.38)	**<0.001**	1.38	(0.87; 2.18)	0.173	0.90	(0.30; 2.72)	0.852	–	–	–
Cough	1.13	(0.44; 2.86)	0.804	3.13	(1.76; 5.59)	**<0.001**	1.79	(1.27; 2.54)	**0.001**	0.79	(0.35; 1.75)	0.554	1.87	(0.46; 7.59)	0.379
Rhinitis	1.60	(0.65; 3.91)	0.308	0.71	(0.37; 3.97)	0.317	1.53	(1.08; 2.19)	**0.018**	1.80	(0.95; 3.42)	*0.073*	0.56	(0.11; 2.91)	0.488
Headache	0.36	(0.04; 3.11)	0.354	0.37	(0.05; 2.93)	0.347	0.48	(0.24; 0.97)	**0.041**	0.56	(0.06; 4.91)	0.603	–	–	–
Abdominal pain	2.98	(0.75; 11.91)	0.122	1.23	(0.38; 3.97)	0.734	0.43	(0.18; 1.00)	*0.050*	2.67	(0.66; 10.71)	0.167	1.48	(0.14; 15.71)	0.745
Nausea/vomiting	0.44	(0.06; 3.48)	0.435	1.60	(0.70; 3.66)	0.269	0.75	(0.42; 1.37)	0.353	1.27	(0.40; 4.01)	0.687	1.07	(0.18; 6.31)	0.939
Diarrhea	0.69	(0.14; 3.28)	0.636	2.01	(0.92; 4.36)	*0.078*	0.54	(0.28; 1.03)	*0.061*	1.46	(0.58; 3.69)	0.423	1.53	(0.29; 8.08)	0.614

*PER, pediatric emergency room; GP, general practitioner; HRV, human rhinovirus. Significant p-values are highlighted in bold.*

## Discussion

This prospective study highlighted a remodeling of the epidemiology of the most common respiratory pathogens in the pediatric population in northern Italy, following the introduction of non-pharmaceutical intervention during the COVID-19 pandemic.

In line with other studies carried out during the pandemic period (Edwards, 2020; Kuitunen et al., 2020; Sullivan et al., 2020; Yeoh et al., 2020; Varela et al., 2021), in the 8 months of the investigation, no case of influenza was recorded and only one case of RSV, although the observation period coincided with the peak of circulation of these viruses (Obando-Pacheco et al., 2018; Puzelli et al., 2019), confirming the efficacy of the non-pharmaceutical interventions. Nevertheless, a peak of HRV positivity was recorded in weeks 40 and 42 of the year, between September and October 2020, and its presence in nasal swabs was constantly detected throughout the period considered in the study.

The most noteworthy data are the total absence of circulation influenza virus and RSV. In our geographical area, no regional surveillance on all pediatric respiratory pathogens is usually provided. Despite this, the Italian Institute of Health, section Epidemiology for Public Health, annually publishes national data relating to the epidemiological surveillance of influenza, which also includes children (Rapporto Influnet, 2018). The available data of influenza syndromes in pediatric population from the period 2017–2018 showed an incidence level of 41.03 cases per 1,000 patients per week in the age range of 0–4 years and of 22.97 cases in the range of 5–14 years, with a peak around the fourth week of 2018. Moreover, previous studies on children from Northern Italy geographical area reported infections of influenza A in 7.6% of children considered and influenza B in 4.4% in the period from 2012 to 2015 (De Conto et al., 2019).

Regarding RSV, although there are no past data on this virus, as identified from hospitalized patients collected and analyzed in the same way, the same study (De Conto et al., 2019) reported it as the most circulating pathogen (27.2%) in pediatric patients with respiratory symptoms in particular in the months between December and March. Another study previously conducted in Italy reported similar data, identifying RSV in 17.2% of enrolled children (Pierangeli et al., 2007). RSV is a major worldwide cause of morbidity and mortality in children under 5 years of age (Mazur et al., 2015), and it has been estimated that almost all children in the world will be infected with RSV by 2 years of age (Glezen et al., 1986). The peak of circulation observed in the Northern Hemisphere occurs between November and March, a period coinciding with that observed in this study (Pangesti et al., 2018).

Despite the unusual absence of influenza and RSV, on the other hand, HRV was consistently detected. It is one of the prevalent viruses of the human respiratory tract and is considered to be the cause of the “common cold” in adults and children, with mostly mild or no symptoms (van Kraaij et al., 2005; Chen et al., 2015). In line with recent European reports (Kuitunen et al., 2020; Poole et al., 2020; Redlberger-Fritz et al., 2021), our data recorded the increase in infections from HRV in conjunction with the reopening of schools, which took place on September in the region considered, for all school grades. Children up to the age of 14 then continued the teaching in presence until the end of the school year (June 2021), while starting from October 2020, the high schools (from 15 years of age) applied a teaching that alternated moments in presence and days in telematic mode. The containment measures, however, provided for the use of face masks and quarantine with the transition to distance learning in the classrooms where positive cases of SARS-CoV-2 were recorded. The peak of HRV positivity is in accordance with the natural circulation of the virus, for which the detection frequency in countries with temperate climates is higher in early fall and, at less extent, in the spring (Gwaltney et al., 1966; Winther et al., 2006; Miller et al., 2007; Piralla et al., 2009).

Questions have been raised as to why HRV is circulating at higher levels than SARS-CoV-2, RSV, and influenza, despite social containment measures. HRV, and other respiratory viruses, can be transmitted through droplets, although it has been shown that while face masks could prevent transmission of human coronaviruses and influenza viruses, they did not reduce the transmission of droplets and aerosols containing rhinoviruses (Leung et al., 2020). Moreover, HRV can persist up to 4 days on inanimate surfaces and can easily reach mucous membranes through contact with contaminated hands, on which it can persist for several hours (Winther et al., 2007; Lee and Gern, 2016). Previous studies described the absence of enveloped viruses and the circulation of non-enveloped viruses such as HRV during the pandemic, raising the hypothesis that non-enveloped viruses are more stable (Takashita et al., 2021). HRV being non-enveloped, unlike the influenza virus, has high resistance to ethanol-based disinfectants; in particular, it has been demonstrated that a single treatment with ethanol hand rub did not reduce the amount of this virus remaining on the skin, increasing the risk of surface contamination (Savolainen-Kopra et al., 2012). Furthermore, it has been speculated that HRV itself may interfere with SARS-CoV-2 infections. *In vitro* studies have shown that HRV infection impairs SARS-CoV-2 replication and spread within the human respiratory epithelium, by triggering an interferon (IFN) response that makes most cells non-permissive to SARS-Cov-2 infection, while on the contrary, HRV is unaffected by the presence of SARS-CoV-2 (Flather and Semler, 2015; Dee et al., 2021). A negative interaction has also been seen between HRV and the flu (Lin et al., 2009; Casalegno et al., 2010; Flather and Semler, 2015; Wu et al., 2020). Although coinfection analysis did not provide significant values, HRV was the most frequent organism that coinfected the same sample. It has been found associated with adenovirus (42.2%), other coronaviruses (16.4%), and bocavirus (12.1%). Regarding the association with SARS-CoV-2, it was found at less extent in 9.5% of the samples, as reported in other studies (Aghbash et al., 2021).

The statistical analysis carried out showed that younger children (<2 years) seemed to be more prone to infections. In particular, SARS-CoV-2, other coronaviruses, and adenovirus were more frequently detected. The current hypotheses focus not only on the lack of a previous immunity (Crowe and Williams, 2003) but also on the lack of the use of the face mask for children under 6 years old, also following the reopening of schools (Salute, 2020a).

Starting from the third week of 2021, an increase in the detection of the other four coronaviruses circulating among humans (229E, HKU-1, NL63, and OC43) was observed. These are viruses commonly spreading in the pediatric population and, unlike other respiratory viruses, do not show a decrease in prevalence with increasing age (Zimmermann and Curtis, 2020). The seasonal pattern of circulation in the Northern Hemisphere occurs between December and April (Vabret et al., 2003; Gaunt et al., 2010). In our study, coronaviruses other than SARS-CoV-2 were detected starting from October 2020, with a maximum between January and February 2021. A recent French study found an increased presence of endemic coronaviruses in younger children during the pandemic, similar to what we have observed (Colson et al., 2020). In this study, the spread of human coronaviruses other than SARS-CoV-2 in the northeast of Italy is taken into consideration for the first time, even in children with moderate respiratory symptoms. These viruses are known to give mild symptoms (Rajapakse and Dixit, 2021), so we speculate on the fact that the peak spread of other coronaviruses recorded in our study, but not of SARS-CoV-2, despite the similar characteristics, could be due to a previous widespread human coronaviruses other than SARS-CoV-2. Furthermore, in the first weeks of 2021, when this increase was recorded, school activity has resumed for many age groups.

Considering the rate of spread of these respiratory pathogens in the pediatric population and their clinical implications, a differential diagnosis seems crucial; this was also highlighted by the statistical models applied, which highlighted that a history of contact with SARS-CoV-2-positive person remains the biggest risk factor for SARS-CoV-2. The only symptom associated with SARS-CoV-2 positivity was fever, which is also associated with adenovirus. These data confirm that COVID-19 symptoms in children are not specific and difficult to distinguish from those associated with other seasonal respiratory diseases (Carlotti et al., 2020; Yasuhara et al., 2020).

In this study, for the first time, a new diagnostic molecular method from nasopharyngeal swabs was introduced for the rapid and simultaneous identification of the main microorganisms, both bacterial and viral, including SARS-CoV-2, associated with infections of the respiratory tract. With the introduction of this test, it was therefore possible to carry out an accurate differential diagnosis, which was essential during SARS-CoV-2 pandemic. Moreover, this technique helped describe the epidemiology of respiratory pathogens during the COVID-19 pandemic in children.

There were some limitations in this study. First of all, this was a monocentric study; however, our hospital is the only maternal–child hospital in the northeast of Italy covering the entire pediatric population of Trieste, with an estimate of more than 31,000 individuals under the age of 18. Second, we cannot compare our results with pre-SARS-CoV-2 data, since no surveillance was carried out in this particular population in the previous years. However, we compared the positivity and the frequency of the different infections with literature data, and similar reduction in influenza and RSV infections have already been described in the literature. Finally, we observed a high variability of the total number of swabs analyzed weekly. However, there were no changes in the referral system for multiplex PCR during the study period. Thus, the observed variability reflects the normal variations of symptomatic acute disease.

In conclusion, our data demonstrated a profound change in the typical epidemiology of pediatric respiratory pathogens during 2020–2021 winter season in a large cohort of children in northeast Italy. Influenza and RSV infections were not detected, whereas HRV was the main pathogen during winter. Social distancing measures, in particular face masks use and school closure, did have an impact on the circulation of common respiratory pathogens. The use of a multiplex PCR allowed a rapid and useful differential diagnosis of common respiratory infections in children during COVID-19 pandemic. Given the novelty of these findings, continuing surveillance for a delayed spread, in particular of RSV and influenza, seems mandatory.

## Data Availability Statement

The original contributions presented in the study are included in the article/supplementary material, further inquiries can be directed to the corresponding author/s.

## Ethics Statement

The studies involving human participants were reviewed and approved by Institutional Review Board (or Ethics Committee) of IRCCS Burlo Garofolo (Protocol Code RC14/2021). Written informed consent to participate in this study was provided by the participants’ legal guardian/next of kin.

## Author Contributions

CC: data curation, writing, and original draft preparation. GZ and LR: formal analysis. DT: data curation. GC, CO, and ADM: investigation. EB: writing, review, and editing. MC: supervision, conceptualization, writing, review, and editing. AA: conceptualization, writing, review, and editing. All authors contributed to the article and approved the submitted version.

## Conflict of Interest

The authors declare that the research was conducted in the absence of any commercial or financial relationships that could be construed as a potential conflict of interest.

## Publisher’s Note

All claims expressed in this article are solely those of the authors and do not necessarily represent those of their affiliated organizations, or those of the publisher, the editors and the reviewers. Any product that may be evaluated in this article, or claim that may be made by its manufacturer, is not guaranteed or endorsed by the publisher.
